# Adenine base editor‐based correction of the cardiac pathogenic *Lmna* c.1621C > T mutation in murine hearts

**DOI:** 10.1111/jcmm.18145

**Published:** 2024-02-08

**Authors:** Luzi Yang, Zhanzhao Liu, Jinhuan Sun, Zhan Chen, Fei Gao, Yuxuan Guo

**Affiliations:** ^1^ School of Basic Medical Sciences Peking University Health Science Center Beijing China; ^2^ Peking University Institute of Cardiovascular Sciences Beijing China; ^3^ Department of Cardiology, Beijing Anzhen Hospital Capital Medical University Beijing China; ^4^ State Key Laboratory of Vascular Homeostasis and Remodeling Peking University Beijing China; ^5^ Beijing Key Laboratory of Cardiovascular Receptors Research Beijing China

**Keywords:** adenine base editor, adeno‐associated virus, bystander effect, DeepABE, inherited cardiomyopathy

## Abstract

Base editors are emerging as powerful tools to correct single‐nucleotide variants and treat genetic diseases. In particular, the adenine base editors (ABEs) exhibit robust and accurate adenine‐to‐guanidine editing capacity and have entered the clinical stage for cardiovascular therapy. Despite the tremendous progress using ABEs to treat heart diseases, a standard technical route toward successful ABE‐based therapy remains to be fully established. In this study, we harnessed adeno‐associated virus (AAV) and a mouse model carrying the cardiomyopathy‐causing *Lmna* c.1621C > T mutation to demonstrate key steps and concerns in designing a cardiac ABE experiment in vivo. We found DeepABE as a reliable deep‐learning‐based model to predict ABE editing outcomes in the heart. Screening of sgRNAs for a Cas9 mutant with relieved protospacer adjacent motif (PAM) allowed the reduction of bystander editing. The ABE editing efficiency can be significantly enhanced by modifying the TadA and Cas9 variants, which are core components of ABEs. The ABE systems can be delivered into the heart via either dual AAV or all‐in‐one AAV vectors. Together, this study showcased crucial technical considerations in designing an ABE system for the heart and pointed out major challenges in further improvement of this new technology for gene therapy.

## 1 INTRODUCTION

Single‐nucleotide variation (SNV) is a major form of genetic aberrations that can cause or modify human diseases. Nearly half of pathogenic SNVs are C•G‐to‐T•A base pair conversions, which can potentially be corrected by the adenine base editors (ABE).^1^ ABEs are ribonucleoprotein complexes that are composed of a single‐guide RNA (sgRNA) and a TadA‐Cas9n fusion protein. TadA is an engineered adenine deaminase that converts adenine into inosine, which is subsequently edited into guanine.^2^ Cas9n is a mutant Cas9 nickase^2^ that locally unwinds the DNA double helix on the sgRNA‐matched genomic locus and expose the target adenine for deamination by TadA. Because ABEs circumvent the adverse and uncontrollable consequences of CRISPR/Cas9‐triggered DNA double strand breaks, they exhibit safer and more precise editing profiles than the conventional CRISPR/Cas9 gene editing.^2^

Cardiovascular diseases (CVDs) are the leading healthcare problems worldwide. ABE provides a novel therapeutic option for CVDs, particularly the ones caused by SNVs. An array of recent studies demonstrated ABE‐based therapy to prevent or reverse inherited cardiomyopathy.^3^, ^4^, ^5^, ^6^, ^7^ Despite these tremendous progress, multiple technical problems remain unsolved. For example, the outcome of ABE editing is highly variable and poorly predictable.^3^, ^4^, ^5^, ^6^, ^7^ Whether a computational model could be harnessed to assess the editing outcome before the expensive experiments were conducted is unclear. Moreover, many TadA and Cas9n variants have been developed for ABE,^2^ but which combinations are more suitable for cardiac gene editing remain undetermined. Designing an ABE system targeting an adenine‐rich region is particularly challenging, as ABE could simultaneously edit multiple adjacent adenines.^8^ How to reduce this bystander effect remains a major problem in ABE applications. Lastly, the canonical ABE systems are oversized and require two adeno‐associated virus (AAV) vectors to deliver to the heart.^3^, ^4^, ^5^, ^6^, ^7^ How to improve this gene delivery system is also a major technical challenge.

Dilated cardiomyopathy (DCM) is a major form of lethal cardiomyopathy that is frequently caused by SNVs in the *LMNA* gene. We recently identified the *LMNA* c.1621C > T mutation in DCM patients and created a knock‐in mouse model carrying this mutation (*Lmna* ^*RC/RC*^ mice).^9^ Based on this model, we attempted to develop an ABE system to correct this mutation in mice. As an orthogonal technical validation, we also demonstrated the design of an ABE system targeting *Camk2d* in the heart. *Camk2d* is a well‐established therapeutic target for many forms of heart diseases including DCM.^10^, ^11^ These efforts generated important new insights regarding the key technical pathways of applying ABEs to the heart.

## 2 RESULTS AND DISCUSSION

**FIGURE 1:**
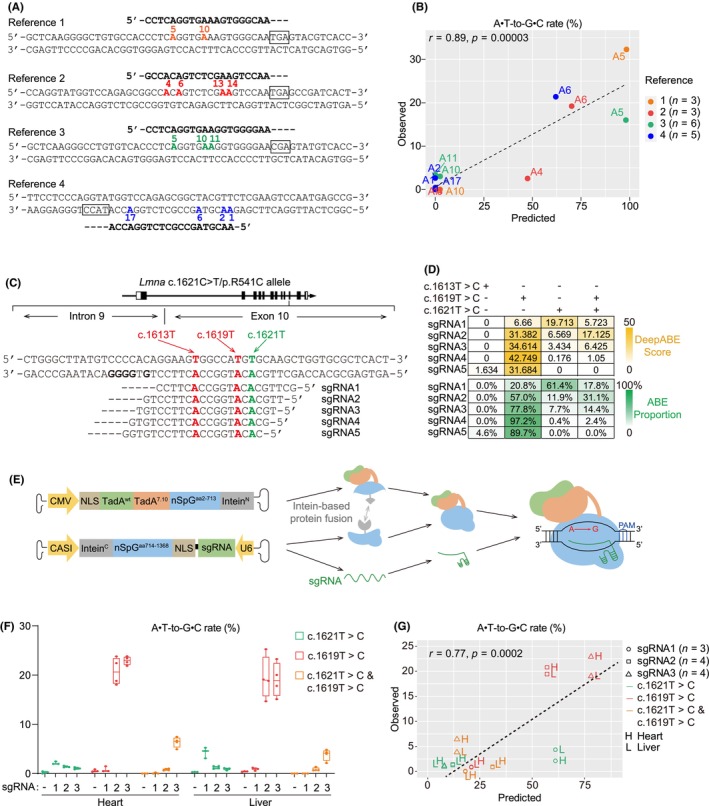
DeepABE‐based prediction of cardiac base editing in mice. (A) A diagram showing adenines that were edited by ABE in the heart in published studies. Adenines were numbered according to their relative distances to the 5′ end of sgRNA. PAM sequences in black boxes. (B) A plot showing predicted versus measured editing rates of each adenine by ABE in (A). Pearson correlation analysis. (C) A diagram showing the genomic locus harbouring the *Lmna* c.1621C > T mutation. Target adenine in green. Bystander adenine in red. (D) DeepABE‐based prediction of editing outcomes for each candidate sgRNA targeting the *Lmna* c.1621C > T mutation. (E) The design and workflow of dual‐AAV‐delivered ABE editing. (F) Amplicon sequencing‐based measurement of ABE editing rates for sgRNA1‐3. (G) A plot showing predicted versus measured ABE editing rates. Pearson correlation analysis. In (B) and (G), n numbers in parenthesis indicate numbers of replicated animals.

Previous studies relied heavily on stem cell or animal models to test if a given adenine can be efficiently edited by ABE.^3^, ^4^, ^5^, ^6^, ^7^ To solve this problem, we tested if DeepABE,^12^ a deep‐learning‐based computational tool, could predict cardiac ABE outcomes in mice. We harnessed published data from four landmark studies using ABE to treat cardiomyopathy in mice^4^, ^5^, ^6^, ^7^ (Figure 1A) and calculated their editing outcomes by DeepABE. The experimental results and the computational prediction exhibit highly robust correlation (Figure 1B).

We next designed sgRNAs targeting the *Lmna* c.1621C > T mutation in mice.^9^ The target c.1621 T base is adjacent to c.1619 T and c.1613 T, which are potential bystanders (Figure 1C). SpCas9‐derived ABEs require a guanine‐containing protospacer adjacent motif (PAM). A guanine‐rich region was found close to the 3′ side of the c.1621 T site, which is suitable for sgRNA design. This fact allowed us to design five sgRNAs (Figure 1C) targeting the c.1621 T base pair and used DeepABE to predict their potential ABE activity. Strikingly, with most sgRNAs, DeepABE implied that ABE would mainly act on c.1619 T and cause a strong bystander effect. SgRNA1 would be the only sgRNA that edits c.1621 T more efficiently than c.1619 T (Figure 1D).

To validate this in‐silico prediction, we constructed dual‐AAV ABE vectors expressing sgRNA1, sgRNA2, or sgRNA3. This system used the constitutively active promoters CMV and CASI to separately express two parts of the TadA7.10‐SpG protein (Figure 1E), which fuse into a full‐length ABE protein via intein‐based trans‐splicing.^13^ TadA7.10 is the prototypic TadA mutant in ABE^1^ while the SpG protein is a mutant SpCas9 with an NGN PAM.^14^

We subcutaneously injected 2 × 10^11^ vg/g (vector genome per gram bodyweight) AAV into postnatal day 1 (P1) *Lmna* ^*RC/RC*^ mice and collected tissues at P7 to assess genome editing results. Targeted amplicon sequencing revealed that sgRNA2 and sgRNA3 mediated up to 20% editing at the c.1619 T site but less than 2% at the c.1621 T site. By contrast, sgRNA1 preferentially triggered c.1621 T editing (Figure 1F). A robust correlation was observed between the predicted and experimental results (Figure 1G), justifying DeepABE as an accountable tool to predict ABE outcomes in the heart.

As an orthogonal validation, we newly designed an array of sgRNAs targeting *Camk2d* (Figure S1), a well‐established therapeutic target for heart diseases including DCM. We designed the sgRNAs to target the Ts in start codon (ATG) or exon‐intron boundaries (the GT motif) so *Camk2d* could be silenced by ABE due to disrupted open reading frames. We predicted ABE editing by these sgRNAs using DeepABE and experimentally measured the actual editing efficiency in Neuro2a cells. We also packaged AAVs expressing two of these sgRNAs and measured editing outcomes in murine hearts. In both experiments, the predicted and experimental data exhibited high correlation (Figure S1B–D). Together, these data testing extra sgRNAs in vitro and in vivo consolidated the conclusion that DeepABE is a reliable tool to predict ABE editing outcomes in the hearts.

It is critical to note from the above experiments that DeepABE does not adjust its prediction according to AAV dosage. The presence of difficult‐to‐transduce cell types such as fibroblasts in the heart also undermines the detectable ABE editing rates. Thus, the experimentally measured ABE editing rates in the heart are always lower than the predicted values by DeepABE. Therefore, a main value of DeepABE is to help us assess the relative editing rates of multiple adenines in the same editing window, nominating easy‐to‐edit sites while reducing the risk of bystander effects.

**FIGURE 2:**
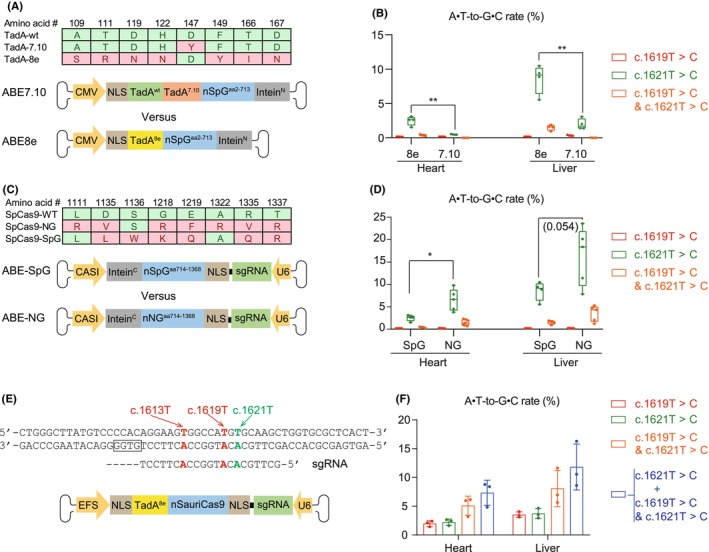
The impact of ABE components and AAV number on editing efficiency. (A) A diagram showing amino acid differences between TadAwt (wildtype), TadA7.10 and TadA8e and the different AAV vectors to deliver TadA7.10‐SpG versus TadA8e‐SpG. (B) The impact of TadA mutants on ABE editing efficiency. (C) A diagram showing amino acid differences between wildtype, NG and SpG versions of SpCas9 and the distinct AAV vectors to deliver TadA8e‐SpG versus TadA8e‐NG. (D) The impact of SpCas9 mutants on ABE editing efficiency. (E) A diagram showing the design of an all‐in‐one AAV vector for ABE‐based correction of the *Lmna* c.1621C > T mutation. (F) Measurement of ABE editing rates using the all‐in‐one vector. In (B) and (D), student's *t*‐test: *p < 0.05; **p < 0.01; non‐significant *p* values in parentheses.

Among all the sgRNAs targeting the *Lmna* c.1621 T site, sgRNA1 exhibited the best ratio of c.1621 T versus c.1619 T editing, therefore the lowest bystander effect (Figure S2). Based on the sgRNA1 system, we next attempted to enhance the editing rate in the heart by modifying TadA. TadA naturally operates as a homodimer. In the original ABE7.10 system,^1^ one wild‐type TadA was fused to one engineered TadA7.10 in tandem to enhance TadA dimerization and therefore the ABE activity. However, when TadA7.10 was evolved into TadA8e^15^ in the following studies, the new TadA8e‐based ABE no longer required two TadAs to fulfill its full capacity. Due to this reason, we next compared an ABE7.10 vector, which includes both a wild‐type TadA and an engineered TadA7.10, versus an ABE8e vector that only included a single TadA8e (Figure 2A).

We injected the same amount of ABE7.10 or ABE8e vectors into *Lmna* ^*RC/RC*^ mice and collected hearts and livers for amplicon sequencing analysis. Interestingly, ABE8e enhanced the editing rate by about 4.9‐fold on the *Lmna* c.1621 T site but not on the c.1619 T site (Figure 2B). We measured the amount of AAV genome in the tissues and observed less AAV genome in the ABE8e group by quantitative real‐time PCR (qPCR), probably due to the variable batch effect of producing different AAV vectors (Figure S3A). This data showed that ABE8e intrinsically exhibited higher editing rate than ABE7.10 on the *Lmna* c.1621 T site.

Next, we compared SpG and NG to determine if changing Cas9 homologues could also modify the gene editing rate by ABE. SpG and NG are two independently developed SpCas9 mutants using the same NGN PAM (Figure 2C).^14^, ^16^ They differ in only seven amino acids, which all locate in the C‐terminal AAV vectors. Thus, we used the same N‐terminus ABE vector in combination with distinct C‐terminus ABE vectors to compare SpG versus NG. We found that the NG‐based ABE editing on the c.1621 T site was 2.5‐fold of the SpG‐based ABE in the heart (Figure 2D). We carefully titrated AAV dosage to ensure the same quantity of AAV genome was transduced into the heart (Figure S3B) and further confirmed the higher editing rate by NG‐based ABE than SpG‐based ABE (Figure S3C). Together, the new TadA8e‐NG combination increased the editing rate on c.1621 T to about 8% in the heart while leaving the bystander effect on c.1619 T at less than 2%.

The dual‐AAV system is unfavored from the standpoint of dosage, side effects, cost and complexity in design. To solve these problems, we next attempted to realize cardiac ABE editing by a single all‐in‐one vector. We examined an array of compact Cas9 homologues and identified the miniature *Staphylococcus auricularis* Cas9 (SauriCas9)^17^ as an ideal tool to construct the all‐in‐one ABE vector. To further reduce the vector size, a small EFS promoter was used to drive SauriABE expression. Conveniently, because the PAM of SauriCas9 (NNGG) is very similar to SpCas9 (NGG), the sgRNAs originally designed for SpCas9 could be directly adopted in SauriCas9 applications (Figure 2E).

We injected 2 × 10^11^vg/g TadA8e‐SauriCas9 vectors, a dose comparable to the ones used in the previous dual‐AAV experiments, into the *Lmna* ^*RC*^ mice. In the heart, we found this all‐in‐one vector resulted in about 2.5% editing rate on c.1621 T alone and about 5% editing rate on c.1621 T and c.1619 T combined (Figure 2F). Thus, the all‐in‐one AAV retained a ~ 8% editing rate (blue bars in Figure 2F) on c.1621 T similar to the dual‐AAV system, but lost the capacity of sgRNA1 to reduce the bystander effect on c.1619 T (Figure 2F). Overall, it is feasible to achieve cardiac ABE editing using an all‐in‐one AAV vector, but the intrinsic properties of the new compact ABE tools might be distinct from the conventional ABE systems, which demands more extensive investigation in the future.

## AUTHOR CONTRIBUTIONS

**Luzi Yang:** Data curation (equal); investigation (equal); methodology (equal); writing – original draft (equal). **Zhanzhao Liu:** Data curation (equal); investigation (equal); methodology (equal). **Jinhuan Sun:** Investigation (equal); methodology (equal). **Zhan Chen:** Methodology (equal); software (equal). **Fei Gao:** Conceptualization (equal); funding acquisition (equal); validation (equal). **Yuxuan Guo:** Conceptualization (equal); data curation (equal); formal analysis (equal); funding acquisition (equal); investigation (equal); resources (equal); validation (equal); writing – original draft (equal).

## CONFLICT OF INTEREST STATEMENT

The authors declare no competing interests.

## Supporting information


Appendix S1.

## Data Availability

Next generation sequencing data are deposited at National Genomic Data Center (accession number: CRA012354, https://ngdc.cncb.ac.cn/gsa/). AAV plasmids will be available at Addgene. Other data supporting the findings of this study are available upon reasonable request.
