# Supplementary material for: Adenine base editor‐based correction of the cardiac pathogenic Lmna c.1621C > T mutation in murine hearts
Source: J Cell Mol Med. 2024 Feb 8;28(4):e18145. doi: 10.1111/jcmm.18145 (PMC10853587; doi:10.1111/jcmm.18145)
Supplement: Supplementary file 1 — Appendix S1. [file JCMM-28-e18145-s001.docx]

# Supplementary Information

# Supplementary materials and methods

## SgRNA design

Candidate sgRNAs were manually designed by identifying PAM sequences adjacent to the target adenine. Then all candidate sgRNAs were assessed by DeepABE at https://deepcrispr.info/DeepBaseEditor. DeepABE score was used as a metrics to predict absolute editing efficiency. DeepABE proportion was used to compare on-target versus bystander adenine editing under the same sgRNA.

Plasmids

Coding sequences for TadA7.10 (Addgene #140002)^1^, TadA8e (Addgene #138491)^2^, SpG (Addgene #140002)^1^, NG (Addgene #138491)^2^ and SauriCas9 (Addgene #189925)^3^ were acquired from Addgene. Split intein-based dual AAV plasmids (Addgene #80930 and #80932)^4^ were used as the backbone to construct all AAV plasmids in this study using the seamless cloning strategy (B632219-0040, BBI, China). Primer synthesis and Sanger sequencing were performed by Beijing Tsingke Biotechnology, China.

## AAV production

AAV production was performed either in house^5^ or with the assistance of PackGene Biotech. In brief, 140 µg AAV vector, 140 µg AAV9-Rep/Cap, and 320 µg pHelper (pAd-deltaF6, Penn Vector Core) plasmids were produced by maxiprep (DP117, Tiangen, China) and triple transfected into HEK293T cells in ten 15-cm plates. 60-72 h after transfection, cells were lysed through by two freeze-thaw cycles to release AAV particles. AAV in culture medium was precipitated by PEG8000 (VWR, 97061-100) and pooled with cell lysates. AAV particles were purified in an Optiprep density gradient (D1556, Sigma) by ultracentrifugation (Beckman Optima XPN-100) with a type 70Ti rotor. The AAV vectors were next concentrated in PBS with 0.001% pluronic F68 (Invitrogen, 24040032) using a 100 kD filter tube (Fisher Scientific, UFC910024).

## Animals

All procedures involving animals were performed in accordance with protocols approved by the Institutional Animal Care and Use Committee of Peking University (approval number LA2021332), and conformed to the Guide for the Care and Use of Laboratory Animals (8th edition. The National Academies Press, 2011) by the Association for Assessment and Accreditation of Laboratory Animal Care. *Lmna^RC/RC^* mice were generated as previously described^6^.

Neonatal *Lmna^RC/RC^* mice were administrated with rAAVs or vehicle subcutaneously under inhalation anesthesia by 2-3% isoflurane (R510-22, RWD, China). During sample collection, mice were euthanized by cervical dislocation. The additional *Camk2d* gene editing experiments were conducted using wild-type C57BL/6J mice.

Cell line

We conducted *Camk2d* ABE editing experiments using the Neuro2a cell line (Procell CL-0168). Cells were cultured in DMEM medium supplemented with 10% FBS and antibiotics, at 37°C with 5% CO_2_. Transfection was performed when the cells reached 70-80% confluency. Firstly, the plasmids to be transfected was prepared in combination with the transfection reagent Lipo8000 (C0533, Beyotime, China) according to the manufacturer's guidelines. Then, the plasmids and transfection reagent complexes were mixed in serum-free Opti-MEM medium. Next, the mixture was slowly added dropwise to the cell culture dish, and gently rocked to ensure even distribution. The cells were then incubated in a 37°C for 48h before samples were collected for analysis.

## Amplicon sequencing

Genomic DNA was extracted from cells and tissues using TIANamp Genomic DNA Kit (DP304, Tiangen, China). The sgRNA-targeted loci containing the *Lmna* c.1621C>T locus or *Camk2d* splicing sites were amplified using Taq PCR MasterMix (KT211, Tiangen, China) and purified by TIANgel Purification Kit (DP219, TIANGEN). See the Supplementary Table for amplicon-seq primer sequences for sequencing library construction (Table S2 and Table S3). Sequencing was performed on an Illumina NovaSeq 6000 platform with 2 × 150 bp pair-end reads at Novogene, China. The sequencing reads were analyzed by CRISPResso2^7^.

Quantitative real-time PCR (qPCR)

Genomic DNA was extracted from heart tissues using the TIANamp Genomic DNA Kit (DP304, Tiangen, China). We detected and quantified the amount of AAV genome in extracted DNA by qPCR. qPCR was performed using Taq Pro Universal SYBR qPCR Master Mix (Q712-02-AA, Vazyme, China), and using primers designed for AAV -specific sequences (Table S1).

## Statistical Analysis

Statistical analysis and plotting were performed using GraphPad Prism. Numbers in parentheses in figures indicate non-significant P-values. Bar plots show mean ± standard deviation. In box plots, horizontal lines indicate the median and 25th and 75th quantiles; whiskers extend to the extreme values.

# Supplementary table

**Table S1. DNA primers for qPCR analysis**

**Table S2. DNA primers for *Lmna* c.1621T amplicon sequencing***

*Barcodes and genome-matching sequences in bold.

**Table S3. DNA primers for *Camk2d* amplicon sequencing***

*Barcodes and genome-matching sequences in bold.

#
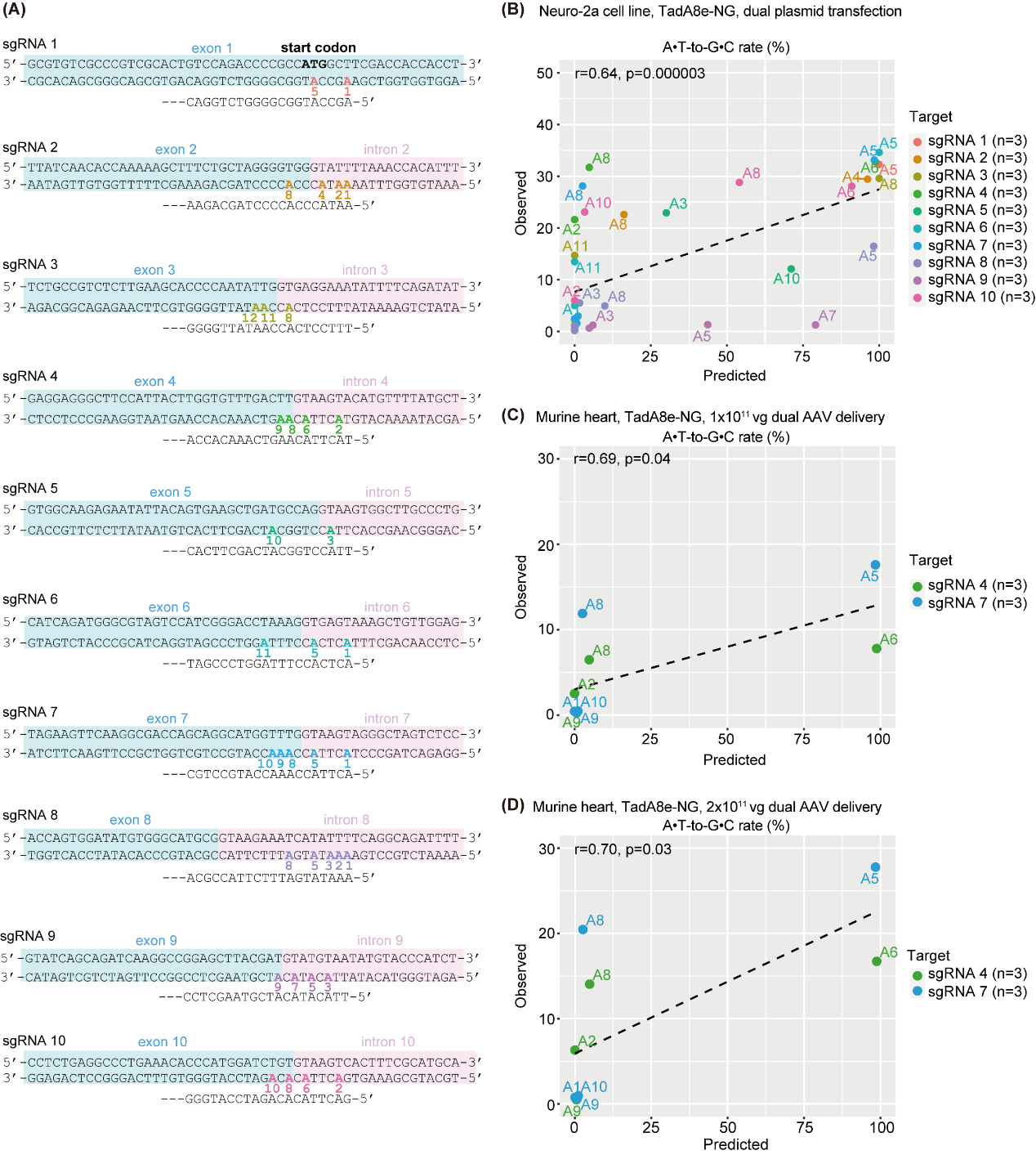
Supplementary figure

**Figure S1. DeepABE validation on additional sites**

**(A)** A diagram showing adenines that were edited by ABE in *Camk2d*. Adenines were numbered according to their relative distances to the 5’ end of sgRNA. **(B)** A plot showing predicted versus measured editing rates of each adenine by ABE in Neuro2a cell culture in vitro. Pearson correlation analysis. **(C-D)** Plots showing predicted versus measured editing rates of each adenine by ABE when mice were injected with 1x10^11^ vg/g **(C)** or 2x10^11^ vg/g **(D)** total AAV in vivo. Pearson correlation analysis.

**
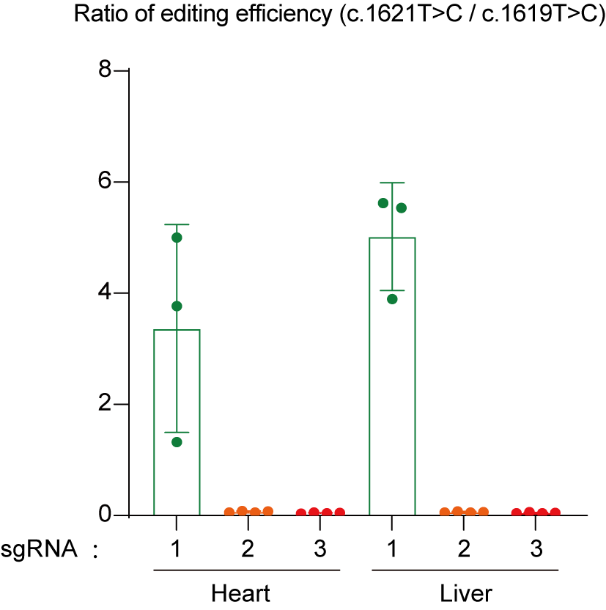
Figure S2. ABE on-target/bystander editing ratio analysis.**

The ratio of editing efficiency between c.1621T>C and c.1619T>C in Figure 1(F).

**
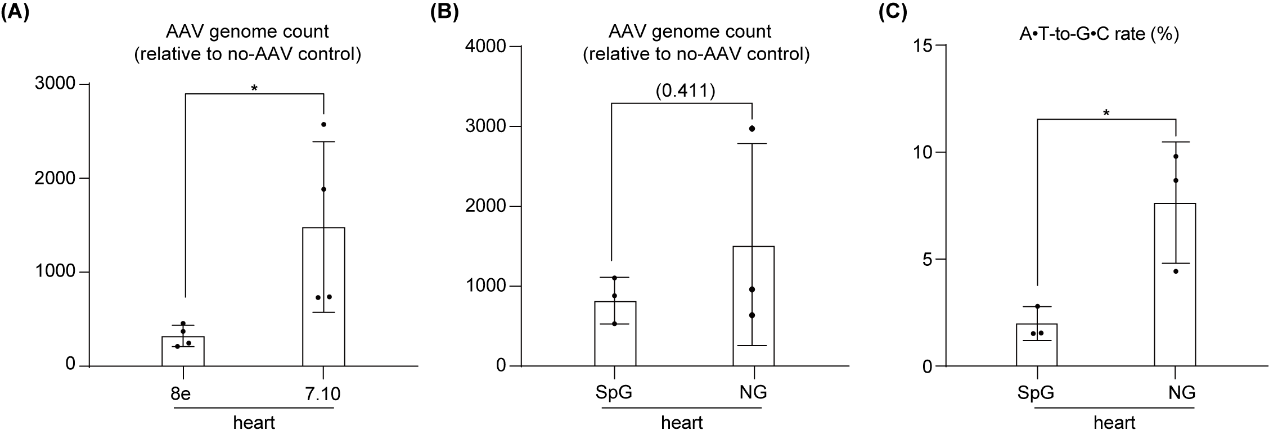
**

**Figure S3. Validation of AAV genome counts in the heart**

**(**A) DNA was extracted from heart samples in the group of ABE8e and ABE7.10 to evaluate the AAV genome counts relative to the no-AAV control by qPCR. **(B)** DNA was extracted from heart samples in the groups of SpG and NG to evaluate the AAV genome counts relative to the no-AAV control. **(C)** When the AAV genome counts are proved equal in SpG versus NG-treated hearts by qPCR, amplicon sequencing reveals the impact of SpCas9 mutations on ABE editing efficiency in the heart. Student’s t-test: *p<0.05; non-significant P values in parentheses.

# References

1 Walton, R. T., Christie, K. A., Whittaker, M. N. & Kleinstiver, B. P. Unconstrained genome targeting with near-PAMless engineered CRISPR-Cas9 variants. *Science (New York, N.Y.)* **368**, 290-296, doi:10.1126/science.aba8853 (2020).

2 Richter, M. F. *et al.* Phage-assisted evolution of an adenine base editor with improved Cas domain compatibility and activity. *Nat Biotechnol* **38**, 883-891, doi:10.1038/s41587-020-0453-z (2020).

3 Davis, J. R. *et al.* Efficient in vivo base editing via single adeno-associated viruses with size-optimized genomes encoding compact adenine base editors. *Nature Biomedical Engineering* **6**, 1272-+, doi:10.1038/s41551-022-00911-4 (2022).

4 Chew, W. L. *et al.* A multifunctional AAV-CRISPR-Cas9 and its host response. *Nature Methods* **13**, 868-+, doi:10.1038/nmeth.3993 (2016).

5 Guo, Y. *et al.* Analysis of Cardiac Myocyte Maturation Using CASAAV, a Platform for Rapid Dissection of Cardiac Myocyte Gene Function In Vivo. *Circ Res* **120**, 1874-1888, doi:10.1161/CIRCRESAHA.116.310283 (2017).

6 Yang, L. Z. *et al.* The LMNA p.R541C mutation causes dilated cardiomyopathy in human and mice. *Int J Cardiol* **363**, 149-158, doi:10.1016/j.ijcard.2022.06.038 (2022).

7 Clement, K. *et al.* CRISPResso2 provides accurate and rapid genome editing sequence analysis. *Nat Biotechnol* **37**, 224-226, doi:10.1038/s41587-019-0032-3 (2019).
